# Peroral Pancreatoscopy for Differentiating Main Duct IPMN from Chronic Pancreatitis: A Narrative Review and a Case Series

**DOI:** 10.3390/diagnostics16162646

**Published:** 2026-08-19

**Authors:** Federica Fimiano, Annachiara De Conte, Carmela Abbatiello, Elio Donnarumma, Mario Gagliardi, Michele Fusco, Giuseppina Pontillo, Mariano Sica, Claudio Zulli

**Affiliations:** 1 Digestive Endoscopy Unit, Gaetano Fucito Hospital, Mercato San Severino, 84085 Salerno, Italy; 2 UOC Clinica Chirurgica e Trapianti di Rene, Dipartimento di Medicina e Chirurgia “Scuola Medica Salernitana”, AOU San Giovanni di Dio e Ruggi d’Aragona, Università degli Studi di Salerno, Via San Leonardo, 84131 Salerno, Italy

**Keywords:** peroral pancreatoscopy, POPS, main-duct IPMN, chronic pancreatitis, endoscopic retrograde cholangiopancreatography, endoscopic ultrasonography

## Abstract

Main pancreatic duct (MPD) dilatation may occur in both main-duct intraductal papillary mucinous neoplasm (MD-IPMN) and chronic pancreatitis (CP). In selected patients, MRI/MRCP, endoscopic ultrasonography (EUS), and contrast-enhanced EUS may remain inconclusive. Peroral pancreatoscopy (POPS) permits direct ductal inspection and targeted biopsy, but the available evidence is heterogeneous and does not establish diagnostic accuracy or clinical utility in the specific differential diagnosis of MD-IPMN versus CP. We performed a narrative review of POPS for suspected pancreatic duct neoplasia and report a retrospective, descriptive, single-center series of five highly selected patients evaluated between January 2023 and November 2024. The case series is presented only to illustrate a multidisciplinary diagnostic pathway; no estimates of accuracy, sensitivity, specificity, or clinical benefit were calculated. All five patients had MPD dilatation > 10 mm, imaging findings compatible with definite or possible chronic pancreatitis, and persistent concern for MD-IPMN after MRI/MRCP, EUS, and contrast-enhanced EUS. POPS-guided biopsies showed low-grade dysplasia in one patient and high-grade dysplasia in two patients. These three patients underwent surgery, and examination of the resection specimens confirmed the same dysplasia categories identified by POPS-guided biopsy. In the remaining two patients, POPS showed regular-appearing ductal epithelium with focal hyperemia, and targeted biopsies revealed nonspecific inflammatory changes. These patients were managed conservatively without surgery. As definitive surgical histopathology was not available, their final diagnostic status remained unconfirmed. Both patients underwent ongoing planned annual surveillance with MRI or EUS and serum CA 19-9 measurement to identify any morphological or biochemical changes. At the most recent follow-up, approximately 192 days (IQR, 116.5–205.5 days) after POPS, no significant changes had been observed. This small, selected series illustrates how POPS may contribute additional visual and histological information when conventional evaluation is discordant. It cannot demonstrate that POPS prevents missed lesions, excludes IPMN, reduces unnecessary surgery, or improves outcomes. Prospective multicenter studies with predefined referral criteria, blinded interpretation, complete reference standards, and long-term follow-up are needed.

## 1. Introduction

Intraductal papillary mucinous neoplasms (IPMNs) of the pancreas are precancerous cystic tumors characterized by proliferation of mucin-producing cells within the main pancreatic duct (MPD) or secondary ducts [1]. IPMNs are classified into three variants (main duct, branch duct and mixed type), and the main duct variant (MD-IPMN) carries the highest malignant potential, with reported malignancy rates ranging from 70% to 92% [1].

Chronic pancreatitis (CP), by contrast, is a progressive fibroinflammatory disease in which destruction of the parenchyma and scar tissue leads to MPD dilatation, calcifications and parenchymal irregularities. The most common symptom of CP is abdominal pain; other manifestations include exocrine pancreatic insufficiency and diabetes, and CP is associated with a high risk of developing pancreatic cancer [2].

Distinguishing MD-IPMN from a dilated MPD in the context of chronic pancreatitis becomes particularly challenging when patients have MPD dilatation > 10 mm together with segmental irregular wall thickening or millimetric mural hyperechoic nodules < 5 mm within the MPD and definite or possible CP criteria. These findings are not clearly suggestive of plugs, calcifications, or intraluminal growth on EUS or contrast-enhanced EUS (CH-EUS). The ability to distinguish neoplastic from inflammatory changes could potentially reduce unnecessary surgical procedures in patients with CP while optimizing surgical candidate selection for IPMN resection.

Misdiagnosis may lead to overly aggressive treatment such as total pancreatectomy or pancreaticoduodenectomy, which is associated with immediate complications (infection, organ failure) and long-term sequelae (diabetes, malabsorption). New endoscopic techniques, such as single-operator POPS, allow direct inspection of the MPD and targeted biopsies, offering a potential tool to address this diagnostic dilemma.

Given these uncertainties, the present study aims to evaluate the role of POPS in the differential diagnosis of CP and MD-IPMN.

## 2. Review Methods

A targeted narrative search of PubMed/MEDLINE and the reference lists of relevant guidelines, systematic reviews, and original studies was conducted. Search terms included combinations of “pancreatoscopy”, “peroral pancreatoscopy”, “single-operator pancreatoscopy”, “SpyGlass”, “slim pancreatoscope”, “eyeMAX”, “intraductal papillary mucinous neoplasm”, “main pancreatic duct”, “chronic pancreatitis”, “adverse events”, “biopsy”, “training”, “learning curve”, “interobserver agreement”, “cost”, and “access”. Studies of pancreatoscopy for pancreatic-duct stones were considered only when they provided information relevant to procedural safety or implementation.

No language restriction was applied during the initial search. Duplicate records were identified by comparison of title, authors, publication year, and digital object identifier and were removed before screening. Because this was a narrative review, no pooled analysis or formal risk-of-bias assessment was performed.

## 3. Clinical Context and Conventional Assessment

### 3.1. Endoscopic Ultrasound (EUS) and Rosemont Criteria for Chronic Pancreatitis

Endoscopic ultrasonography remains the most sensitive examination for early chronic pancreatitis. The Rosemont criteria standardize EUS findings into major and minor categories [3]:

- **Major criteria**—hyperechoic foci with shadowing and intraductal calculi (Major A); lobularity with honeycombing (Major B) [3].
- **Minor criteria**—lobularity without honeycombing, hyperechoic foci without shadowing, MPD dilatation and irregular contour, cysts, hyperechoic strands, dilated side branches, or an echogenic MPD margin [3].
- **Classification**—a Major A feature plus ≥ 3 minor features indicates definite chronic pancreatitis; Major A with <3 minor features or Major B with ≥3 minor features suggests chronic pancreatitis; 3–4 minor features or Major B with <3 minor features defines indeterminate cases, while ≤2 minor features are considered normal [3].

Although EUS provides high sensitivity (81–84%) for early diagnosis [3], inflammatory changes may overlap with neoplastic lesions. Calcifications, irregular contours and hyperechoic foci and dilation of the MPD may resemble the intraductal nodules of IPMN, and the absence of pathognomonic signs makes exclusion of MD-IPMN difficult.

### 3.2. Imaging and Criteria for MD-IPMN

International (Kyoto) guidelines [4] define “high-risk stigmata” for IPMN as: (1) MPD diameter ≥ 10 mm; (2) enhancing mural nodules ≥ 5 mm or a solid component; (3) obstructive jaundice; and (4) suspicious or positive cytology. “Worrisome features” include enhancing mural nodules < 5 mm, thickened enhancing cyst wall, elevated tumor markers, acute pancreatitis, abrupt change in pancreatic duct caliber with distal pancreatic atrophy. Contrast-enhanced EUS and MRCP are often used to confirm the presence of MPD nodules.

However, in patients with definite or possible chronic pancreatitis and MPD dilatation, MPD segmental irregular wall thickening, small (<5 mm) hyperechoic nodules may appear that are difficult to distinguish from inflammatory plugs.

### 3.3. Single-Operator Pancreatoscopy

#### 3.3.1. Principles and Technique

In recent years, POPS has emerged as a promising complementary diagnostic tool. Using dedicated endoscopes such as the SpyGlass^®^ System (Boston Scientific, Natick, MA, USA), direct visualization of the main pancreatic ductal mucosa can be achieved, allowing for targeted biopsies and acquisition of morphological data essential for diagnosis and surgical planning [5,6,7].

The procedure is performed through a therapeutic duodenoscope; pancreatic sphincterotomy is recommended to facilitate SpyGlass introduction. Usually the examination starts in the tail and progresses towards the head. Contraindications include active cholangitis and MPD diameter < 5 mm [1].

The European Society of Gastrointestinal Endoscopy recommends prophylactic antibiotic use in advanced biliopancreatic endoscopic procedures [8]. However, the use of prophylactic antibiotics specifically in POPS remains highly debated.

#### 3.3.2. Next-Generation Slim-Diameter Pancreatoscopes

Recently, slim-diameter digital cholangiopancreatoscopes have been introduced as potential alternatives to conventional single-operator systems such as SpyGlass DS. The 9-Fr eyeMAX system (Micro-Tech, Nanjing, China) has a smaller outer diameter, a highly maneuverable distal section, a dedicated irrigation channel, and a 1.1 mm working channel that permits targeted biopsy. Its reduced diameter may facilitate passage through the papilla and navigation within the pancreatic duct, potentially limiting the extent of pancreatic sphincterotomy required. In addition, compatibility with endoscopes equipped with a 3.2 mm working channel may expand its use in patients with surgically altered anatomy [9].

In an initial pancreatobiliary series, POPS using the 9-Fr eyeMAX was technically successful in 12 of 13 patients, and tissue sampling was achieved in most procedures, with no adverse events reported in the POPS subgroup [9]. Nevertheless, these findings derive from small, retrospective, highly selected cohorts. The narrower working channel may restrict accessory selection and biopsy size, while comparative data regarding image quality, maneuverability, histological yield, durability, and adverse events remain limited. Moreover, biopsy–resection concordance was imperfect, and no prospective head-to-head comparison with SpyGlass DS has established diagnostic or safety superiority. These devices should therefore be regarded as promising emerging platforms requiring further independent prospective evaluation rather than as established replacements for conventional systems.

### 3.4. Pancreatoscopic Features of IPMN

POPS allows observation of villous, vegetative or “fish-egg-like” projections, mucin, visible tumor vessels, mucosal friability and wall thickening [1]. The “fish-mouth papilla” sign (bulging papilla with mucin extrusion) is considered pathognomonic but has limited sensitivity (25–68%) [1]. Hara’s classification describes five types of lesions based on their endoscopic appearance and correlates them with dysplasia grade [10]. Types I and II are predominantly benign (hyperplasia or low-grade lesions), while Types III–V are more frequently associated with high-grade dysplasia or invasive carcinoma. Lesion height and vascularization show a progressive increase from Type I to Type V, correlating with malignant potential [10] (Table 1).

In a study of 60 patients with confirmed IPMN, no carcinomas were found in Types 1 and 2, whereas Types 3–5 were malignant; diagnostic accuracy for main-duct IPMN was 88%, with specificity of 78% and sensitivity of 68% [10].

### 3.5. Pancreatoscopic Features of Chronic Pancreatitis

In chronic pancreatitis, MPD strictures are common because of persistent inflammation and fibrosis [1]. Thickened and friable mucosa is commonly observed in malignant strictures. In contrast, in the absence of a true stricture within the setting of chronic pancreatitis, the ductal mucosa appears congested, thickened, hyperaemic and dystrophic, without protruding wall projections or nodules [10,11].

### 3.6. Clinical Evidence

Table 2 summarizes representative evidence. Reported ‘clinical impact’ generally means that management changed after POPS; it does not prove that the change improved outcomes or avoided inappropriate surgery. Accuracy estimates are especially vulnerable to selection bias, incomplete reference standards, and incorporation of the index test into the final diagnosis.

## 4. Studies on Pancreatoscopy for IPMN

A meta-analysis has shown that POPS offers sensitivity of 64–100% and specificity of 75–100% for IPMN diagnosis [12], providing additional information not detected by radiological imaging in about 42% of cases [1]. Endoscopic mapping of lesion extent changed the surgical plan in 14–60% of patients [1].

A prospective Japanese multicenter study (n = 27 patients with IPMN and mural nodules) evaluated POPS using the SpyGlass DS system to guide surgical decision-making. Under POPS-DS guidance, biopsy specimens were adequate for histologic assessment in all patients with main-duct mural nodules and in 67% of those with branch-duct mural nodules; fluid specimens collected near the target mural nodules were also adequate for cytology in every case. Because conventional sampling in branch-duct lesions may miss mural nodules, combining POPS-guided biopsy with pancreatic juice cytology is advisable. The authors concluded that POPS can help select patients for resection and avoid unnecessary surgery [15].

## 5. EUS-FNA Cytology: Diagnostic Yield for Malignancy in PCLs

In the preoperative work-up of pancreatic cystic lesions (PCLs), EUS-guided fine-needle aspiration (EUS-FNA) cytology primarily serves as a rule-in test for malignancy. In a recent systematic review and meta-analysis by Ding et al. (15 studies; 755 patients; reference standard: surgical histology), pooled sensitivity for malignancy was 0.62 (95% CI 0.42–0.78) and pooled specificity 0.96 (95% CI 0.91–0.98). Sample adequacy was high but imperfect (pooled 0.85), consistent with the known limitations of cytology related to cellularity and sampling. Overall, these data confirm that a positive cytology strongly supports malignancy, whereas a negative result does not reliably exclude it, reinforcing the need for complementary diagnostic strategies in clinically equivocal cases [16].

## 6. Pilot Study with Next-Generation Sequencing

A 2025 prospective study evaluated 22 patients with a dilated main pancreatic duct (mean diameter 12.4 mm) who underwent EUS-guided aspiration of ductal fluid for differential diagnosis between MD-IPMN and chronic pancreatitis [17]. Only the fish-mouth papilla sign (observed in 3 of 12 MD-IPMN cohort, i.e., 25%) reliably distinguished MD-IPMN from chronic pancreatitis; other clinical features, cytology, CEA and KRAS analysis failed to discriminate [17]. Indeed, analysis of cyst fluid CEA obtained by EUS-guided aspiration shows a sensitivity of 73% and a specificity of 84% [18]. In contrast, EUS-FNA–based cytology has a pooled sensitivity of 0.63 (95% CI 0.56–0.70) and specificity of 0.88 (95% CI 0.83–0.93) for the diagnosis of mucinous pancreatic cystic lesions [19], while KRAS analysis was not discriminatory.

In the molecular analysis, GNAS mutations were detected exclusively in 11 of 12 (91.6%) MD-IPMN patients and were absent in chronic pancreatitis, whereas KRAS mutations were present in both conditions. The authors suggest that combining genetic markers with standard diagnostics may improve discrimination. However, EUS-guided tissue sampling for pancreatic cystic neoplasms has historically been avoided in Japan because of concerns that EUS-guided interventions might disseminate neoplastic cells more frequently in cystic lesions than in pancreatic solid tumors. In a nationwide Japanese survey, the estimated rate of neoplastic cell dissemination from EUS-guided fine-needle aspiration of pancreatic solid tumors was ~0.33% [20].

## 7. Safety and Limitations

POPS requires expertise and adequate MPD dilatation. The most frequent complication of POPS is post-procedural pancreatitis. In the meta-analysis by Saghir et al. evaluating POPS for pancreatic duct stones (16 studies; 383 patients), pooled adverse event rates were as follows: post-ERCP pancreatitis (PEP) 7.0% (95% CI 3.5–13.6; I^2^ = 38%), fever 3.7% (95% CI 2.0–6.9; I^2^ = 0), abdominal pain 4.7% (95% CI 2.7–7.8; I^2^ = 0), perforation 4.3% (95% CI 2.1–8.4; I^2^ = 0), and haemorrhage 3.4% (95% CI 1.7–6.6; I^2^ = 0); no procedure-related mortality was reported [21].

In the meta-analysis by De Jong et al., the pooled adverse event (AE) rate was 12% (95% CI 9–17%). Post-ERCP pancreatitis (PEP) was the most frequent complication, with a pooled incidence of 10% (95% CI 7–15%). When reported, PEP severity was mild in 70.6% of cases, moderate in 20.6%, severe in 5.9%, and unknown in 2.9%; one patient with severe PEP died. Other AEs included post-sphincterotomy bleeding (1.3%), a mild sedation-related event (3.2%), and cholangitis (8.3%) [12].

Beyond adverse events, POPS has several practical limitations that restrict its routine use. Successful ductal access generally requires sufficient MPD dilatation, while a narrow duct, tight stricture, marked duct angulation, intraductal debris, or difficult papillary access may prevent advancement of the pancreatoscope or complete inspection of the target segment. Although slim-diameter devices may facilitate access, their potential technical and safety advantages have not yet been established in comparative trials.

POPS also requires advanced ERCP expertise and has a procedure-specific learning curve involving stable ductal cannulation, atraumatic advancement, irrigation, recognition of mucosal patterns, and accurate targeted biopsy. Dedicated equipment and compatible biopsy accessories are not universally available, and acquisition, maintenance, and single-use device costs may limit implementation outside high-volume referral centers. Formal cost-effectiveness analyses are currently lacking.

Finally, interpretation of pancreatoscopic findings remains partly subjective. Morphological features such as villous projections, fish-egg-like lesions, abnormal vessels, friability, and inflammatory mucosal changes may overlap, and the available classification systems have undergone limited external validation. Evidence concerning interobserver agreement is particularly limited for pancreatoscopy. Standardized terminology, structured image documentation, dedicated training, and prospective assessment of interobserver agreement are therefore required before visual impressions can be applied consistently across centers.

In patients with chronic pancreatitis who undergo POPS for pancreatic duct stone removal, the procedural complication rate is typically estimated to remain below 5–10% [22,23]. However, when POPS is performed for the evaluation of pancreatic tumors, the risk of complications rises to approximately 12% [12].

Limitations include duct caliber, the presence of tight strictures and difficulty maneuvering in side branches. Inadequate mucosal clearance can reduce visibility; therefore, the duct should be cleaned with a balloon, and the procedure performed cautiously.

## 8. Accessibility, Cost, Learning Curve, and Interobserver Variability

Accessibility is limited by the need for advanced ERCP expertise, dedicated processors or controllers, disposable scopes and biopsy forceps, anesthesia or deep sedation resources, pathology support, and rapid access to management of complications. Direct acquisition costs vary by country and procurement contract. Published cost-effectiveness analyses largely concern biliary cholangioscopy, not diagnostic POPS; therefore, no claim of cost-effectiveness can be made for POPS in MD-IPMN versus CP. POPS is categorized as an advanced cholangiopancreatoscopy procedure in the 2025 ESGE position statement. Training should occur in high-volume referral centers after competence in ERCP has been established, with structured supervision and outcome monitoring [24].

## 9. Case-Series Methods

### Design, Setting, and Reporting Purpose

This was a retrospective, descriptive, single-center case series of five consecutive patients who underwent POPS during routine clinical care between January 2023 and November 2024. These patients represented the entire eligible cohort, and all five were included in the present case series. The predefined eligibility criteria had been established before patient inclusion, and all patients fulfilled these criteria. Thus, the eligible denominator was five, with no eligible patients excluded. The series was assembled to illustrate the diagnostic pathway in highly selected patients with discordant findings. It was not designed as a diagnostic-accuracy study, and no control group, sensitivity, specificity, predictive values, or estimates of clinical utility were used.

Predefined eligibility criteria were age ≥ 18 years; MPD diameter ≥ 10 mm; segmental irregular duct-wall thickening and/or intraductal protrusions < 5 mm; definite, suggestive, or indeterminate CP features according to the Rosemont framework; MRI/MRCP and EUS/contrast-enhanced EUS that did not resolve the differential diagnosis; and an HPB multidisciplinary decision that a specific question could reasonably be addressed by POPS. Predefined exclusion criteria were a definitive management plan independent of POPS; active acute pancreatitis or infection; contraindication to ERCP, anesthesia, or antithrombotic interruption; duct anatomy or caliber judged unsuitable; inability to provide clinical consent; or unavailable records. Pancreatoscopic findings were interpreted jointly in real time by two endoscopists during the procedure. Because the assessments were performed jointly rather than independently, interobserver agreement was not evaluated.

All POPS procedures were performed as part of routine clinical care, based on individual clinical indications and multidisciplinary assessment, and not for research purposes. Ethics Committee approval covered the retrospective collection and analysis of clinical data generated before approval, including data from procedures performed from January 2023 onward.

## 10. Results

Our cohort included five patients who underwent EUS between January 2023 and November 2024 for the differential diagnosis of chronic pancreatitis (CP) versus suspected main-duct IPMN (MD-IPMN). All patients underwent contrast-enhanced abdominal MRI with MRCP and EUS, which showed main pancreatic duct (MPD) dilatation > 10 mm with either segmental irregular mural thickening or millimetric hyperechoic mural nodules (<5 mm). These findings were not clearly suggestive of mucin plugs, calcifications, or intraluminal tumor growth on EUS or contrast-enhanced EUS (CH-EUS). The pancreatic parenchyma also displayed features consistent with CP according to the Rosemont criteria. CH-EUS showed variable enhancement. Given the persistent diagnostic uncertainty, POPS with the SpyGlass DS system was scheduled. POPS enabled direct high-definition visualization of the pancreatic ductal epithelium and targeted biopsy of abnormal-appearing mucosal lesions.

**Figure 1:**
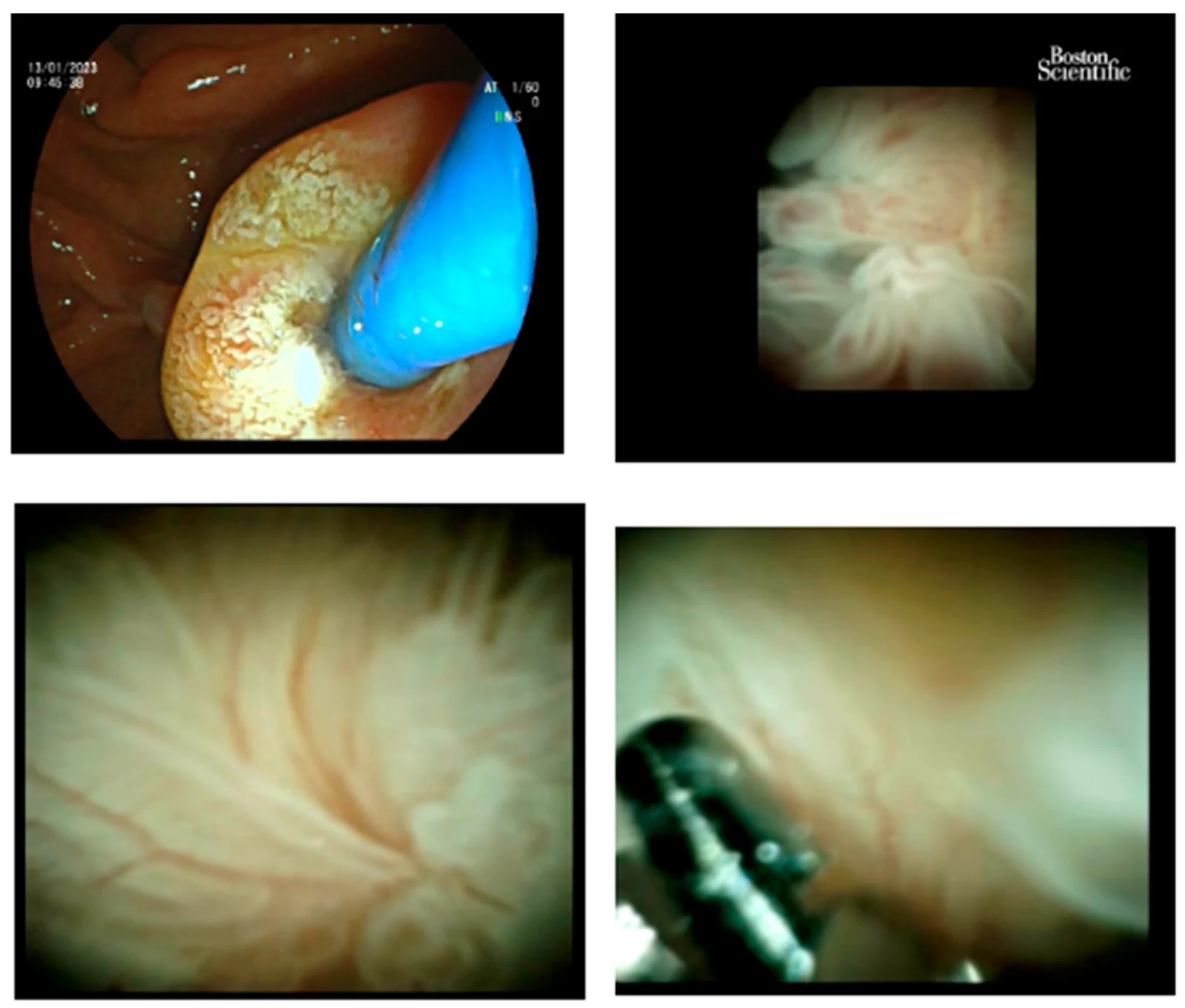
Representative institutional POPS images showing villous-type protrusions within the main pancreatic duct, consistent with Hara type 4 morphology. Images are included only after written permission/consent for publication of anonymized endoscopic images has been confirmed.

The cohort had a mean age of 60 years (3 males and 2 females) and a median follow-up of 192 days. Mean MPD dilatation was 17.2 mm (SD, 7.5 mm) (Table 3). In two cases, POPS revealed an MPD covered by regular epithelium with raised areas of hyperemia, and SpyBite biopsies showed nonspecific inflammation. In one patient, the MPD was covered by fish-egg-like protrusions with vascular structures (Hara type 3 morphology [10]), while in two cases it showed villous-type protrusions (Hara type 4 morphology [10]) (Figure 1), with histology confirming low-grade IPMN in one patient and high-grade IPMN in two. The three patients with IPMN underwent surgery after multidisciplinary team discussion, with histology confirming low-grade dysplasia in one and high-grade dysplasia in two. Two patients with inflammatory biopsies were managed without surgery and underwent MRI or EUS follow-up. These observations indicate only that no neoplasia was demonstrated in the sampled tissue; they do not exclude MD-IPMN elsewhere in the duct. Their final diagnostic status therefore remains unconfirmed pending longer documented surveillance.

### Adverse Events and Follow-Up

No procedure-related adverse events were observed. The median follow-up was 192 days (IQR, 137.5–205.5 days).

## 11. Discussion

Differentiating main-duct intraductal papillary mucinous neoplasm (MD-IPMN) from chronic pancreatitis remains clinically challenging, as both entities may present with main pancreatic duct (MPD) dilation and segmental duct-wall thickening. Although endoscopic ultrasound (EUS) is highly sensitive for chronic pancreatitis, EUS findings alone cannot always reliably distinguish inflammatory thickening of the MPD wall from neoplastic involvement related to IPMN.

In this context, EUS-guided sampling and cytology may be limited by indeterminate results and imperfect diagnostic performance, and biomarkers such as carcinoembryonic antigen (CEA) measured in pancreatic fluid—while relatively specific for mucinous pathology—often lack sensitivity. These limitations have driven interest in integrating molecular diagnostics: genetic alterations (e.g., GNAS mutations) detected in ductal aspirates are being explored to improve discrimination between IPMN and chronic inflammatory disease and to better refine risk stratification [17].

POPS may provide additional visual and histological information by enabling direct inspection of the ductal lumen, including assessment of villous projections, vascular patterns, and mucin, and by allowing targeted biopsies [1]. Previous studies have reported diagnostic performance estimates and changes in surgical decision-making in selected patients; however, these findings do not establish improved clinical outcomes and should be interpreted in light of selection bias and incomplete reference standards [14].

In our institutional series, POPS-guided biopsies identified dysplasia in three of five highly selected patients with persistent diagnostic uncertainty after conventional imaging. These observations illustrate the additional findings obtained through direct ductal visualization and targeted biopsy. Given the descriptive design, small sample size, absence of a comparator, and incomplete reference standard in the two nonsurgical patients, this series cannot establish improved diagnostic accuracy, reclassification of disease status, clinical utility, or better patient management.

Nevertheless, our case series has important limitations, most notably its small sample size and the single-center, highly selected nature of the cohort, which constrain generalizability. Two authors have consultancy relationships with Boston Scientific, the manufacturer of the SpyGlass DS system used in all five procedures. Although the company had no role in study design, patient selection, data collection or analysis, interpretation of the findings, manuscript preparation, or the decision to submit the article, these relationships may represent a potential source of positive evaluation or interpretation bias. Larger prospective studies are needed to define the optimal clinical and imaging thresholds for POPS referral and to clarify how best to integrate endoscopic, cytologic, biochemical, and molecular data into a standardized diagnostic algorithm.

## 12. Conclusions

In this single-center case series with narrative review, POPS was used as an adjunct in five selected patients with persistent uncertainty between CP-related ductal changes and suspected MD-IPMN. The observations suggest that POPS may help characterize selected equivocal MPD lesions by combining direct intraductal visualization with targeted biopsy.

However, these results must be interpreted in light of important limitations. Our experience derives from a small, single-center case series with a limited sample size and inherent selection bias toward complex, ambiguous cases. Larger, prospective multicenter studies are therefore warranted to define optimal clinical and imaging thresholds for POPS referral and to determine how best to integrate endoscopic, cytologic, biochemical, and molecular data into a standardized diagnostic algorithm.

## Figures and Tables

**Table 1:** Hara classification of protruding lesions.

Hara Type	Endoscopic Appearance	Reported Histological Association
Type 1	Granular mucosa	Usually benign/low-grade; malignancy not confirmed in the original classification series
Type 2	Fish-egg-like protrusions without vascular structures	Predominantly low-grade lesions
Type 3	Fish-egg-like protrusions with vascular structures	Reported association with carcinoma in situ or invasive cancer
Type 4	Villous projections	Higher malignant potential
Type 5	Vegetative projections	Higher malignant potential

**Table 2:** Representative studies evaluating diagnostic peroral pancreatoscopy.

Author/Year	Study Design	Patients/Procedures	Indication	Diagnostic Performance	Adverse Events	Major Conclusions
Hara et al., 2002 [10]	Retrospective surgical cohort	60 patients	Characterization and grading of IPMN by POPS and IDUS	Visual classification accuracy: 88% for MD-IPMN and 67% for BD-IPMN.	Overall AEs: 4/60 (6.7%) in the systematic-review extraction [12].	The five-type morphological classification helped distinguish noninvasive from malignant IPMN; vascularized, villous, and vegetative lesions were associated with more advanced pathology.
Nagayoshi et al., 2014 [6]	Retrospective single-center cohort	17 patients	Suspected IPMN with MPD dilatation; direct visualization and tissue/fluid sampling	Targeted biopsy: sensitivity 25%, specificity 100%. Irrigation-fluid cytology: sensitivity 100%, specificity 100%.	Overall AEs: 6/17 (35%) in the systematic-review extraction [12].	First-generation SpyGlass enabled direct inspection and sampling. Irrigation-fluid cytology complemented biopsy, but estimates arose from a very small selected cohort.
de Jong et al., 2023 [12]	Systematic review and meta-analysis	25 studies	Diagnostic characterization and preoperative mapping of suspected IPMN	Across eligible studies: sensitivity 64–100%, specificity 75–100%, and overall diagnostic accuracy 87.5–100%.	Pooled overall AE rate 12% (95% CI 9–17%); pooled post-ERCP pancreatitis rate 10% (95% CI 7–15%).	POPS is usually technically feasible and may improve diagnosis and surgical mapping, but evidence is heterogeneous and affected by selection, verification, and publication bias.
El Hajj et al., 2017 [13]	Retrospective single-center cohort	79 patients; 102 procedures	Suspected pancreatic-duct neoplasia or indeterminate ductal abnormalities	Visual impression: sensitivity 87%, specificity 86%, accuracy 87%. Visual assessment plus targeted sampling: sensitivity 91%, specificity 95%, accuracy 94%.	12/102 procedures (12%).	POPS had high technical success and provided useful visual and histological information. Interpretation is limited by heterogeneous indications and reference standards.
Vehviläinen et al., 2022 [14]	Primarily retrospective, three-center cohort	101 procedures	Preoperative evaluation of suspected main-duct or mixed-type IPMN	Formal diagnostic accuracy against an independent reference standard was not reported; management was affected in 86/101 cases (85%).	Post-POPS pancreatitis: 20/101 (20%); one fatal case.	POPS frequently influenced management and identified alternative causes of MPD dilatation, but clinical impact should not be interpreted as proof of diagnostic accuracy or improved outcomes.

**Table 3:** Patient-level clinical, imaging, POPS, pathology, management, and follow-up data.

Case	Age/Sex	Lifestyle: Alcohol Consumption (≥2 Units/Day) or Smoking	MRI/MRCP	EUS/CH-EUS	Reason for POPS	POPS Appearance	Targeted Biopsy	Adverse Events	Surgery/Pathology	Management & Follow-Up	Final Diagnostic Status
P1	50 yo, M	Alcohol consumption and smoking	MPD 30 mm; segmental irregular wall thickening	Rosemont major + minor criteria	Discordant CP features and concern for MD-IPMN	Regular epithelium with focal hyperemia	Nonspecific inflammation	None	No surgery	MRI surveillance at 6 months and then every 9–12 months. Follow-up: 150 days.	Unconfirmed; nonspecific inflammatory changes; surveillance ongoing.
P2	57 yo, F	Alcohol consumption	MPD 18 mm; segmental irregular wall thickening	Rosemont minor criteria	Discordant CP features and concern for MD-IPMN	Regular epithelium with focal hyperemia	Nonspecific inflammation	None	No surgery	MRI/EUS surveillance at 6 months and then every 9–12 months. Follow-up: 192 days.	Unconfirmed; nonspecific inflammatory changes; surveillance ongoing.
P3	71 yo, F	Smoking	MPD 13 mm; segmental irregular wall thickening	Rosemont minor criteria	Characterize visible intraductal abnormality and obtain tissue	Fish-egg-like protrusions with visible vascular structures (Hara type 3 morphology)	IPMN, low-grade dysplasia	None	IPMN with low-grade dysplasia; margin R0	Surgery (pancreaticoduodenectomy) after MDT discussion. Follow-up: 202 days.	Definitive: resected IPMN with low-grade dysplasia.
P4	60 yo, M	Alcohol consumption	MPD 13 mm; segmental irregular wall thickening	Rosemont minor criteria	Characterize visible intraductal abnormality and obtain tissue	Villous projections (Hara type 4 morphology)	IPMN, high-grade dysplasia	None	IPMN with high-grade dysplasia; margin R0	Surgery (pancreaticoduodenectomy) after MDT discussion. Follow-up: 209 days.	Definitive: resected IPMN with high-grade dysplasia.
P5	62 yo, M	Alcohol consumption	MPD 12 mm; segmental irregular wall thickening	Rosemont minor criteria	Characterize visible intraductal abnormality and obtain tissue	Villous projections (Hara type 4 morphology)	IPMN, high-grade dysplasia	None	IPMN with high-grade dysplasia; margin R0	Surgery (pancreaticoduodenectomy) after MDT discussion. Follow-up: 83 days.	Definitive: resected IPMN with high-grade dysplasia.

## Data Availability

The original contributions presented in this study are included in the article. Further inquiries can be directed to the corresponding author.

## Abbreviations

AE, adverse event; CEA, carcinoembryonic antigen; CH-EUS, contrast-harmonic endoscopic ultrasound; CP, chronic pancreatitis; EUS, endoscopic ultrasound; HGD, high-grade dysplasia; IPMN, intraductal papillary mucinous neoplasm; LGD, low-grade dysplasia; MD-IPMN, main-duct intraductal papillary mucinous neoplasm; MPD, main pancreatic duct; MRI, magnetic resonance imaging; MRCP, magnetic resonance cholangiopancreatography; MDT, multidisciplinary team; PCL, pancreatic cystic lesion; PEP, post-ERCP pancreatitis; POPS, peroral pancreatoscopy.
